# Surface display of OmpC of *Salmonella* serovar Pullorum on *Bacillus subtilis* spores

**DOI:** 10.1371/journal.pone.0191627

**Published:** 2018-01-25

**Authors:** Xixi Dai, Minggang Liu, Kangcheng Pan, Jinlong Yang

**Affiliations:** 1 Chongqing Academy of Animal Science, Rongchang, Chongqing, China; 2 College of Veterinary Medicine, Sichuan Agricultural University, Chengdu, Sichuan, China; 3 Fujian Luodong Bio-Technology Co., Ltd., Putian, Fujian, China; National Institute of Animal Biotechnology, INDIA

## Abstract

*Salmonellosis* is a major public health problem throughout the world. Thus, there is a huge need for diversified control strategies for *Salmonella* infections. In this work, we have assessed the potential use of *Bacillus subtilis* (*B*. *subtilis*) spores for the expression of a major protective antigen of *Salmonella* serovar Pullorum, OmpC. The expression of OmpC on the surface of spores was determined by immunofluorescence microscopy. Mice immunized with recombinant spores expressing the OmpC antigen presented significant levels of OmpC-specific serum IgG and mucosal SIgA antibodies than in mice immunized with non-recombinant spores (*p*<0.01). In addition, oral immunization with recombinant spores was able to induce a significant level of protection in mice against lethal challenge with *Salmonella* serovar Typhimurium. These results suggest that *B*. *subtilis* spores have promising potential in the development of mucosal vaccines against *Salmonella* infections.

## Introduction

Pullorum disease (PD) is a worldwide poultry disease that caused enormous economic losses throughout large areas of the world. *Salmonella* serovar Pullorum (*S*. Pullorum) is the etiological agent of PD, which is an acute septicemic disease that results in anorexia, diarrhea, dehydration, weakness and high mortality in chicks and poults, and loss of weight, decreased egg production and hatchability, diarrhea, and lesions and abnormalities of the reproductive tract in mature fowl [1, 2]. Although different strategies to control *Salmonella* infections are currently available, continuous emergence of multi-drug resistance [3, 4] and novel *Salmonella* variants [5, 6] are still a mortal threat to the poultry industry and public health. Thus, it is urgently needed to develop new control strategies for PD in poultry.

Since most pathogenic organisms, including *Salmonella*, penetrate to the host through the mucosal membranes, effective mucosal vaccines that induce immunity at the site of infection are able to confer more efficient protective immune responses against *Salmonella* [7, 8]. There is now much evidence that OmpC, an outer membrane protein (porin) from *Salmonella*, is a promising candidate antigen that efficiently stimulates innate and adaptive immune responses [9, 10]. However, without suitable antigen delivery systems and adjuvants, most protein antigens are poorly immunogenic when mucosal immunization [11].

*B*. *subtilis* is a non-pathogenic aerobic Gram-positive and endospore-forming bacterium that generally regarded as safe (GRAS) [12]. *B*. *subtilis* spores, an extremely stable form under harsh life conditions, are covered with a multilayered coat, which composed of at least 70 different protein species [13]. Previous studies have confirmed that spore coat protein can be employed as fusion partner for expression and display of vaccine antigens on the spore surface, and protective systemic and mucosal immune responses were elicited following oral or intranasal administration of recombinant *B*. *subtilis* spores without adjuvants [14–18]. Moreover, the spores presenting antigens also have the ability to induce both antigen specific CD4+ and CD8+ T cell cellular immune responses [18–20]. Excellent resistance properties and safety, together with several other attractive advantages, such as a readily genetic manipulation, low production cost, and easy administration, transport and storage, make *B*. *subtilis* spore an ideal candidate for the expression and delivery of vaccine antigens to complex and rigorous mucosal surfaces where antigens are sampled.

In the present study, we successfully constructed a recombinant *B*. *subtilis* spores expressing immunogenic antigen OmpC of *S*. Pullorum on the spore surface. Mice were orally immunized with recombinant spores to evaluate the mucosal and systemic antigen-specific immune responses. In addition, the protective efficacy of recombinant spores was also investigated in mice using a challenge experiment. This work indicates that spore-based expression and delivery system of vaccine antigens, as a novel strategy, has promising future in protection of mucosal surfaces against invasion by *Salmonella*.

## Materials and methods

### Ethics statement

All animal experiment procedures were carried out in strict accordance with the recommendations of the Guide for the Care and Use of Laboratory Animals of the Chinese Association for Laboratory Animal Sciences (http://www.calas.org.cn/). The Institutional Animal Care and Use Committee (IACUC) at the Sichuan Agricultural University approved all the procedures used in this study (Protocol NO. DKY-S20123517).

### Mice

Female BALB/c mice (specific pathogen-free, SPF) aged approximately 6–8 weeks were obtained from the Vital River, Beijing, China. Mice were raised under SPF conditions at 24±2°C with a light-controlled regimen (12-hour light/dark cycle). Mice were anesthetized intraperitoneally with ketamine (40 mg/kg), and all efforts were made to minimize suffering. In the challenge experiment, mice were closely monitored for signs of *Salmonella* infection, and mice that developed clear clinical symptoms or any signs of infections include diarrhea, fever, and abdominal cramps were humanely euthanized.

### Bacterial strains

*S*. Pullorum strain CVCC533 and *S*. Typhimurium strain SL1344 were issued by China Institute of Veterinary Drugs Control (Beijing, China). The integrative vector pDG364 and *B*. *subtilis* strain 168 (*trp*^*‾*^) were obtained from Bacillus Genetic Stock Center (BGSC) (Columbus, OH). The subcloning vector pMD19-T and *Escherichia coli* strain DH5α were purchased from TaKaRa (Dalian, China).

### Construction of integrative recombinant plasmid

**Fig 1:**
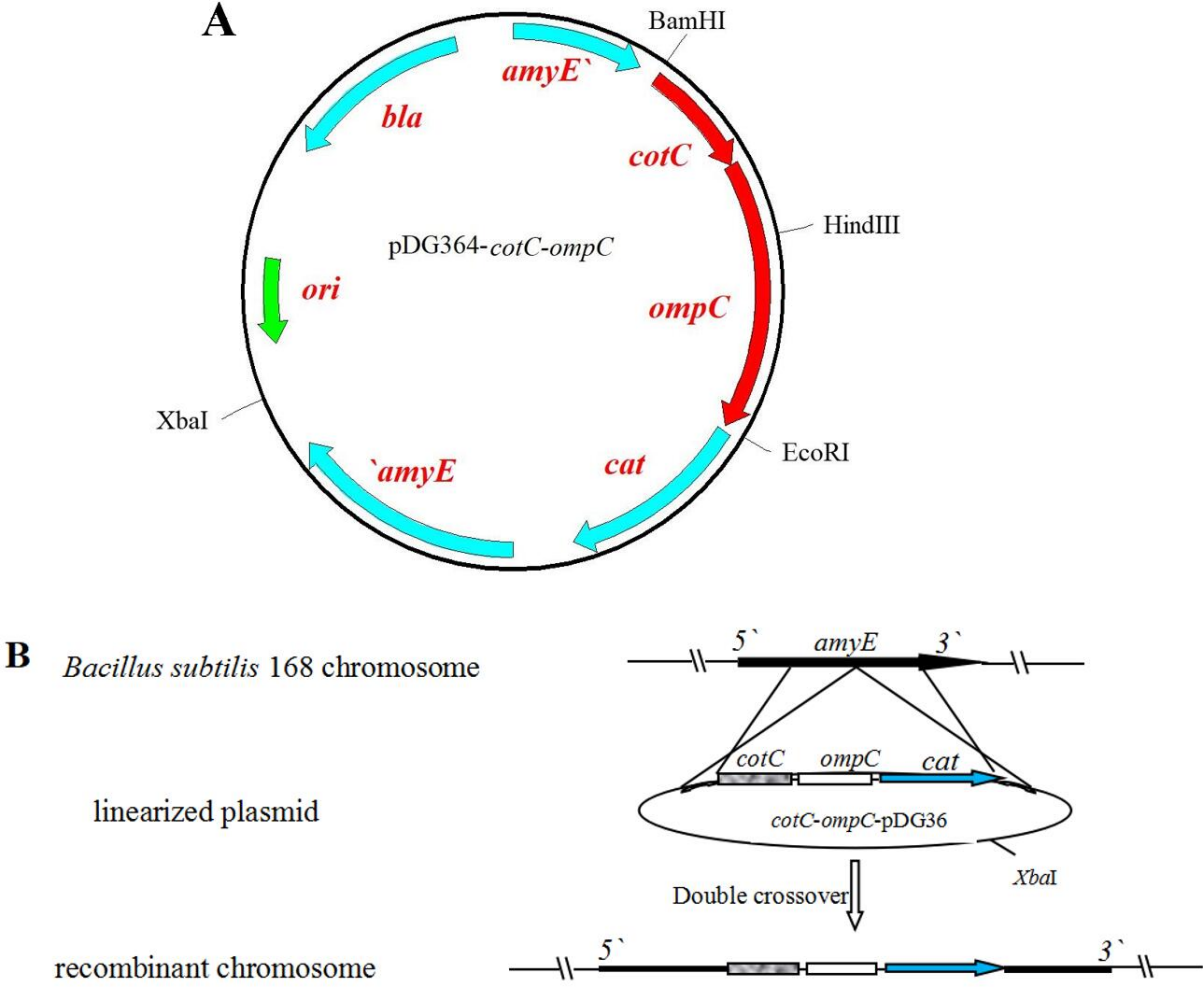
Schematic representation of the construction of the recombinant spores. The *cotC*::*ompC* gene fusion and *cat* (chloramphenicol-resistance gene) gene carried by plasmid pDG364-*cotC*-*ompC* were integrated into the *amyE* gene locus of *B*. *subtilis* 168 chromosome by double cross-over recombination events. Arrows indicate direction of transcription.

The integrative recombinant plasmid was constructed by introducing *cotC*::*ompC* gene fusion into the pDG364 (Fig 1A). Firstly, a 1078 bp DNA fragment coding for OmpC (GenBank accession NO. CP_003047) was PCR amplified using *S*. Pullorum chromosome as a template and oligonucleotides ompC-F and ompC-R as primers (Table 1). The PCR product was sequentially digested with *Hind*Ⅲ and *Eco*RI and cloned into the pDG364 previously digested with the same enzymes, yielding plasmid pDG364-*ompC*. A purified *cotC* gene (GenBank accession NO. NC_000964) containing the promoter sequence and the whole coding sequence was amplified by PCR using the *B*. *subtilis* chromosomal as template and oligonucleotides cotC-F and cotC-R as primers (Table 1). The PCR product was sequentially digested with *Bam*HI and *Hind*Ⅲ and cloned in frame to the 5‵ end of the *ompC* gene carried by plasmid pDG364-*ompC*, yielding plasmid pDG364-*cotC*-*ompC*.

**Table 1:** Oligonucleotides list.

Name	Sequence *^a^*	Restriction site
cotC-F	CGGGATCCTGTAGGATAAATCGTTTGGGC	*Bam*HI
cotC-R	GGGGGGGAAGCTTGTAGTGTTTTTTATGCTTTTTATAC	*Hind*Ⅲ
ompC-F	CCCAAGCTTAATAAAGACGGCAACAAATTAGACC	*Hind*Ⅲ
ompC-R	CGAATTCTTAGAACTGGTAAACCAGACCC	*Eco*RI
amyE-F	CCAATGAGGTTAAGAGTATTCC	Null
amyE-R	CGAGAAGCTATCACCGCCCAGC	Null

^*a*^The underlined letters indicate the introduction of restriction sites.

### Chromosomal integration

Plasmid pDG364-*cotC*-*ompC* was linearize by digestion with *Xba*I and used to transform competent cells of the *B*. *subtilis* strain 168 under previously described procedures [21]. Chloramphenicol-resistant recombinant *B*. *subtilis* were obtained by double-crossover recombination event (Fig 1B), and several clones for each transformation were tested by amylase activity analysis [21] and then confirmed by PCR using chromosomal DNA as template and four oligonucleotides (ompC-F and ompC-R, amyE-F and amyE-R, amyE-F and ompC-R, ompC-F and amyE-R) (Table 1) to prime the reaction.

### Immunofluorescence microscopy

The expression and display of fusion protein CotC-OmpC on the spore surface was confirmed using immunofluorescence microscopy, about 10 μl of spore suspension was prepared and fixed on slides as methods adapted from previous report [22, 23]. Samples were blocked for 30min with 3% (w/v) bovine serum albumin (BSA) in PBS (pH7.4) at room temperature (RT) and then washed ten times with PBS. The samples were incubated overnight at 4°C with OmpC antiserum (raised in chicken), washed ten times and then incubated further with Cy3-labeled goat anti-chicken IgG (Sangon Biotech, 1:2000 in PBST) for 45 min at RT. After washing procedures, samples were viewed and photographed under fluorescent microscope (Eclipse, TE2000U, Nikon).

### Immunization in mice

A total of 120 mice were randomly divided into 5 groups (24 for each): Group A and group B were dosed orally (0.2 ml) with spores of either recombinant *B*. *subtilis* or isogenic wild-type *B*. *subtilis* 168 by gavage (1.0×10^10^ CFU spores/time/ mouse) at 0, 15 days respectively; group C and group D were administrated orally by feeding the diet mixed with spores of either recombinant *B*. *subtilis* or isogenic wild-type *B*. *subtilis* 168 (1.0×10^6^ CFU spores/g); a naïve, untreated control group was also included (group E).

### Indirect ELISA for detection of OmpC-specific serum and mucosal antibodies

On 1 day before and 22 days after the first dose, mice from each group were anesthetized with an intraperitoneal ketamine injection at 40 mg/kg body weight, bled retro-orbitally, and sacrificed by cervical dislocation. Intestinal content samples were immediately collected from each mouse in the sterile operation. Samples were tested by ELISA as previously described [24–26] with the following modifications: Flat-bottom microtiter plates (high-binding capacity; Sangon Biotech, China) were coated with 100μl of purified *S*. Pullorum strain CVCC533 (1×10^9^ CFU/ml) in carbonate buffer solution (pH9.6) overnight at RT. After the plates were blocked for 1h at 37°C with 3% BSA, serum samples were applied as a 1/50 dilution in diluted buffer. Replicate samples were utilized together with a negative control (pre-immune serum). After incubation at 37°C for 1h, the plates were washed and then incubated with HRP-conjugate rabbit anti-mouse IgG (Santa Cruz Biotechnology). Plates were incubated for 1h at RT and then developed using TMB substrate. Reactions were stopped using 2M H_2_SO_4_, and optical densities (ODs) were read at 450 nm.

ELISA of small intestinal content samples was tested with a similar method, and IgA was detected by using HRP-conjugate goat anti-mouse IgA (Santa Cruz Biotechnology).

### Protection studies

22 days after the first dose, 16 mice of each group mentioned above were randomly chosen and divided into two groups and were challenged with approximately 4×10^8^CFU (2×LD_50_) and 2×10^9^CFU (10×LD_50_) [27]of *S*. Typhimurium strain SL1344 by the intra-peritoneal rout, respectively. The animals were strictly monitored 72h after challenge, and individuals showing clear clinical symptoms or any signs of *S*. Typhimurium infections include diarrhea, fever, and abdominal cramps were considered unprotected and humanely euthanized by overdose of ketamine.

### Statistical analysis

Samples were tested individually, and results were expressed as the arithmetic mean ± S.D. (standard deviation). Statistical significance of the difference between group means was assessed by unpaired Student’s *t*-test, and the significance level was set at *P* ≤0.05. Statistical analysis was performed with SPSS for Windows version 16.0.

## Results

### Construction and chromosomal integration of *cotC*::*ompC* gene fusion

To obtain recombinant *B*. *subtilis* spores expressing OmpC on their surface, a recombinant plasmid pDG364-*cotC*-*ompC* containing the *cotC*::*ompC* gene fusion for double cross-over with *B*. *subtilis* chromosome was constructed by fusing the *ompC* gene into frame of the coding part of *cotC* gene (Fig 1A). And then *cotC*::*ompC* gene fusion was integrated into the *B*. *subtilis* chromosome at the nonessential *amyE* gene locus by double cross-over event (Fig 1B)

**Fig 2:**
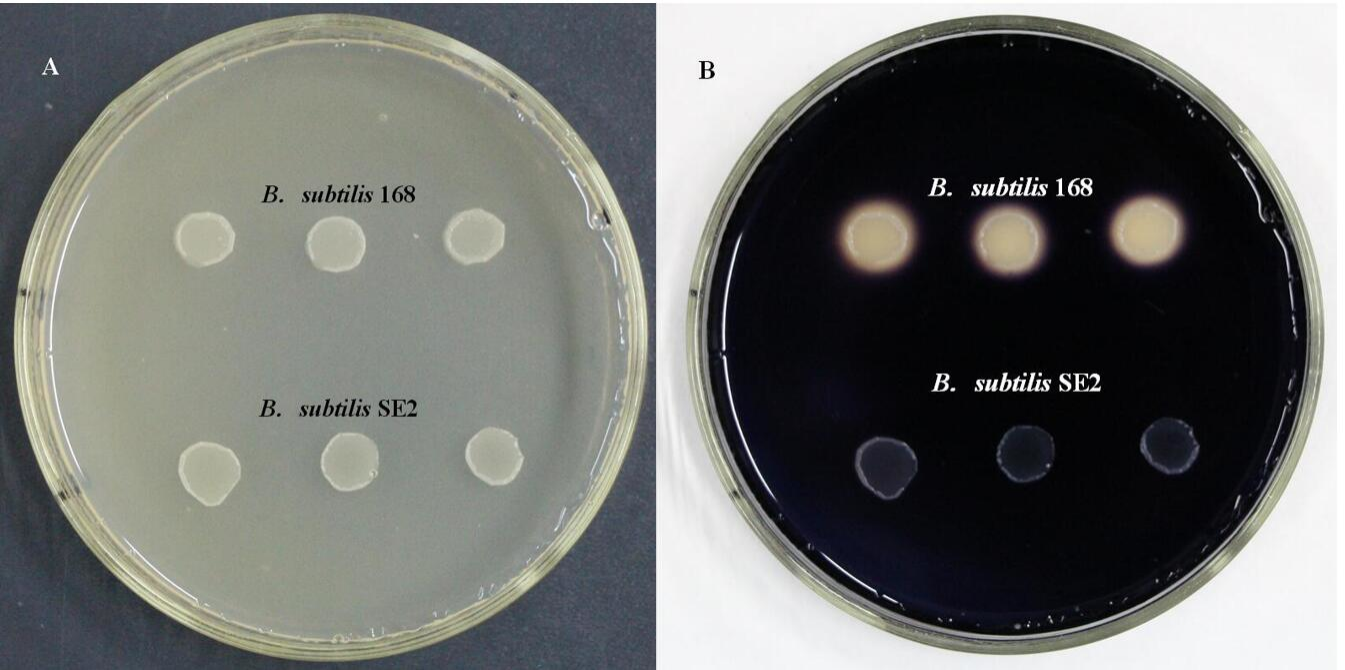
Amylase activity analysis. Recombinant and nonrecombinant strains grew on the starch-containing LB plate before (A) and after (B) being stained by iodine. The integration of *cotC*::*ompC* gene fusion disrupts *amyE* gene and made the strain amylase deficient, transparent halo was produced around the *B*. *subtilis* 168, but in the recombinant *B*. *subtilis* SE2 clones, no transparent halo was produced.

Individual clones for each transformation were first tested by amylase activity analysis and named *B*. *subtilis* SE2. Integration of *cotC*::*ompC* gene fusion at the *amyE* locus can interrupt the expression and secretion of amylase. As a result, no white halo was noted around the recombinant clones on a starch-containing plate stained by iodine (Fig 2).

**Fig 3:**
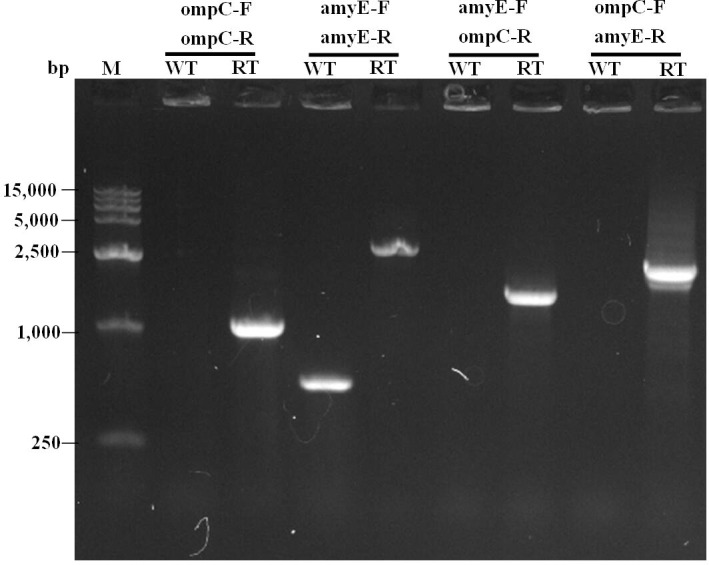
PCR analysis with different primer pairs. Site-directed PCR analysis of *cotC*::*ompC* gene fusion integrated into the chromosome of *B*. *subtilis* 168. lane WT, *B*. *subtilis* 168; lane RT, recombinant *B*. *subtilis* SE2; primer pairs used in PCR are labeled above.

To further verify that *cotC*::*ompC* gene fusion were localized at the *amyE* locus, individual clones for each transformation were tested by PCR using different primer pairs. Primers amyE-F and amyE-R amplified a 556 bp wild-type fragment and a 2,500 bp integrated fragment (Fig 3). Moreover, primers ompC-F and ompC-R, amyE-F and ompC-R, ompC-F and amyE-R produced fragments around 1,078 bp, 1,600 bp, and 1,700 bp using the recombinant *B*. *Subtilis* SE2 chromosome as a template, respectively. However, no PCR products were observed in wild-type *B*. *Subtilis* chromosome (Fig 3). All these results above indicated that the occurrence of correct chromosomal integration of *cotC*::*ompC* gene fusion.

### Surface display of OmpC on the recombinant spores

**Fig 4:**
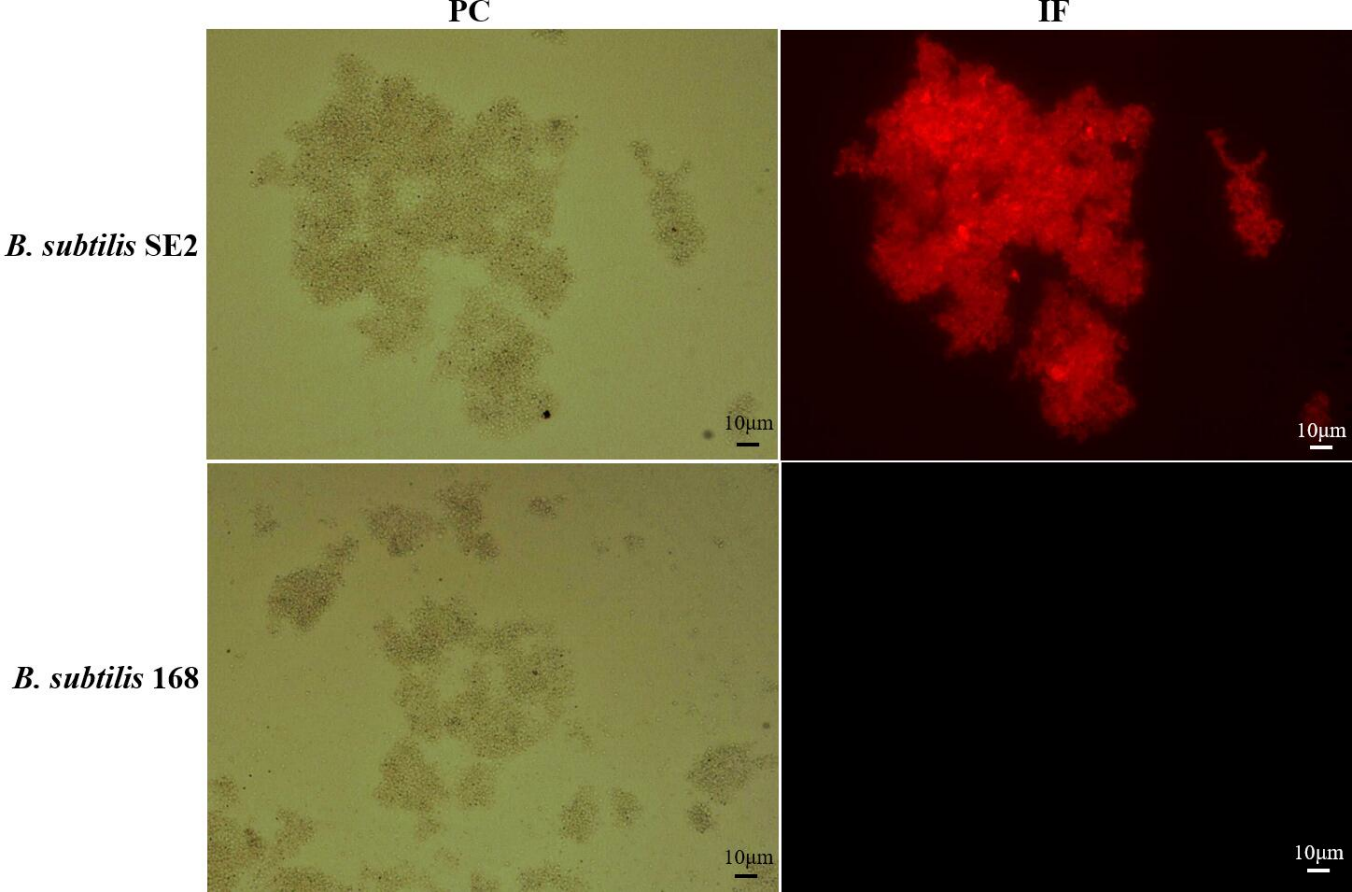
Immunofluorescence microscopy analysis. Sporulation of *B*. *subtilis* strains was induced by the exhaustion method, and spores were collected in DSM after 48h. Spores were labeled with OmpC antiserum followed by Cy3-labeled goat anti-chicken IgG. Spores were visualized by phase-contrast (PC) and immunofluorescence (IF) microscopy.

To verify the expression of OmpC antigen on the surface of the spores, intact spores purified from wild-type and isogenic recombinant strains were probed by fluorescence immunoassay with OmpC-specific primary antibodies and Cy3-labeled goat anti-chicken IgG secondary antibodies. Distinctive red fluorescent staining was detected on the recombinant *B*. *subtilis* SE2 spores surface (Fig 4). However, no fluorescence signal was detected on the surface of wild type *B*. *subtilis* 168 spores (Fig 4). The results indicated that OmpC was successfully expressed on the surface of the recombinant spores.

### Oral delivery of the OmpC displayed on spores induces systemic and mucosal antibodies responses

**Fig 5:**
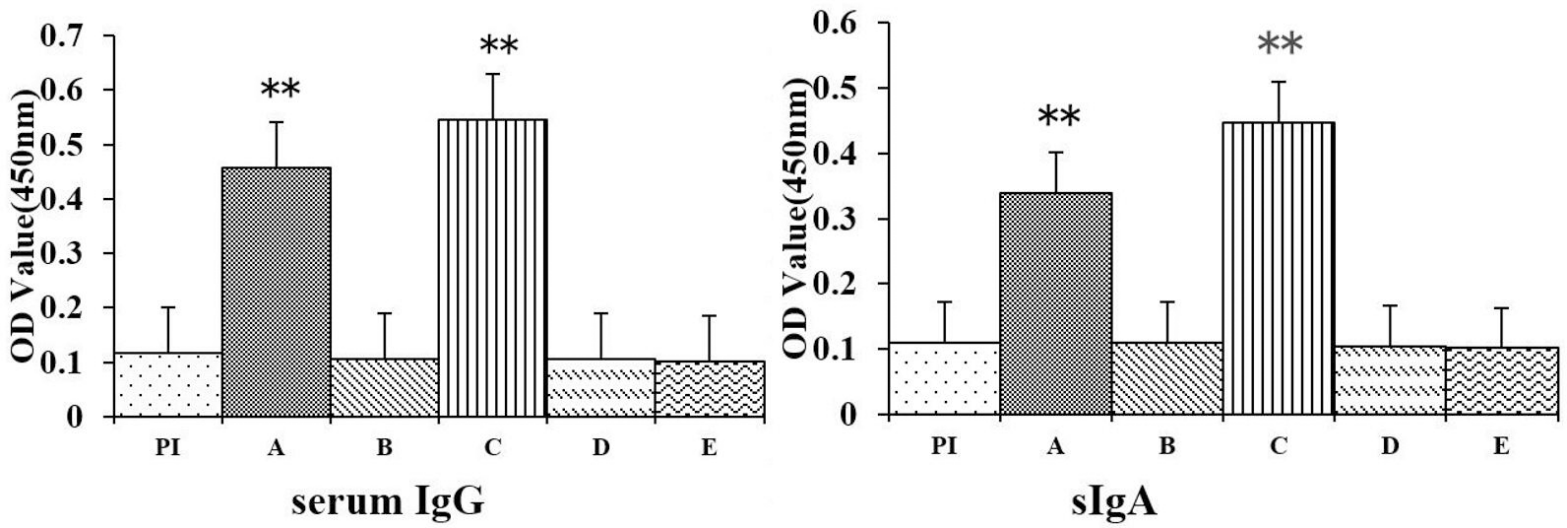
Serum IgG and intestinal mucosal SIgA antibodies responses. PI, pre-immune samples. Samples obtained from the mice immunized with recombinant spores (A and C) and wild-type spores (B and D); E, samples obtained from a naïve, untreated control group. ** indicates *p*<0.01.

To evaluate the immune responses derived by recombinant spores administrated by different immunization strategies, serum and small intestinal content samples were collected from mice before immunization and 22 days after the first dose and were tested by ELISA. The levels of both OmpC-specific serum IgG and intestinal mucosal SIgA antibodies in mice dosed with recombinant *B*. *Subtilis* SE2 spores (group A and C) were significantly higher (*p*<0.01) than those of mice dosed with nonrecombinant spores (group B and D), or the naïve control group (group E) (Fig 5). Moreover, mice dosed by feeding the diet mixed with recombinant spores (group C) presented significant levels of anti-OmpC serum IgG and intestinal mucosal SIgA responses (*p*<0.05) than mice dosed by gavage with recombinant spores of the same strains (group A) (Fig 5).

### Protection in mice

In order to assess the protection potential of recombinant *B*. *subtilis* spores, mice immunized with spores were challenged with *S*. Typhimurium strain SL1344 by the intra-peritoneal route as described in Material and Methods. The results presented here show that mice orally dosed with recombinant spores (group A and C) can induce 100% protection against a challenge dose of 2×LD_50_, and when mice were challenged with 10×LD_50_, only 50% protection was observed in the group A and 75% protection was observed in the groups C (Table 2). In comparison, 100% mortality occurred in the mice immunized with wild type *B*. *subtilis* spores (group B and D) and all naïve mice (group E) (Table 2).

**Table 2:** Protection of mice against challenge with *S*. Typhimurium strain SL1344.

Groups	2×LD_50_		10×LD_50_	
	Dead/Total No.	Protection	Dead/Total No.	Protection
A	0/8	100%	4/8	50%
B	8/8	0	8/8	0
C	0/8	100%	2/8	75%
D	8/8	0	8/8	0
E	8/8	0	8/8	0

## Discussion

Since *Salmonellae* are facultative intracellular pathogens that are capable of subverting host immune defenses by adopting various strategies [28], prevention and elimination of *Salmonella* have become the enormous challenge. Thus, to develop new mucosal candidate vaccines by employing more immunogenic and protective components of *Salmonella*, such as outer membrane proteins, is a significant issue. The immune response of the host is usually directed initially to surface-associated components of the bacterial cell [29]. In addition, recent evidence indicates that surface-associated antigens are promising antigens candidates for the induction of both humoral and cellular immunity to *Salmonella* [30]. The porin OmpC of *Salmonella* was identified as the major surface antigen with unique exposed immune epitopes, and can induce both innate and adaptive immunity in the host [9, 10, 31]. Moreover, the nucleotide sequence analysis of different *Salmonella* serotypes indicates that *ompC* gene is highly conserved [32, 33]. Thus, OmpC has considerable potential in development of vaccines against mucosal infection by different *Salmonella* serovars.

However, protein antigens are poorly immunogenic when delivered by oral immunization due to enzymatic or chemical degradation in the gastro-intestinal tract. *B*. *subtilis* spores are extremely resistant to environmental stresses, such as degradation in the protease-rich condition of gastro-intestinal tract, and oral immunization with spores carrying vaccine antigens can induce mucosal and systematic immune response in the absence of adjuvants [14, 15]. In this study, we have shown that the outer coat component CotC of *B*. *subtilis* spores can be used as a fusion partner for surface display of OmpC on the spore surface. Mice orally immunized with recombinant *B*. *subtilis* SE2 spores were able to stimulate appreciable anti-OmpC serum IgG and intestinal mucosal SIgA responses. The local SIgA immune response observed in this study is critical and necessary for mucosal immunity against invasion by *Salmonella*, and demonstrates that spores have a potential to be developed into an effective delivery system to enhance protective immune responses at mucosal surfaces.

Moreover, a striking observation from this work is that oral immunization with recombinant spore expressing OmpC of *S*. Pullorum was able to confer a significant level of protection in mice against lethal challenge with *S*. Typhimurium. The most likely reason for this cross-protection activity is that the pore-functioning porins of *Enterobacteriaceae* are highly conserved in the course of evolution and have similar biological structure and immunogenicity [34, 35]. There is no doubt that spore-based vaccine antigen delivery systems are still in their infancy. Despite several unique advantages, as compared to other delivery systems, the low expression efficiency of vaccine antigens by recombinant spores is a major obstacle for spore-based vaccine development. Many efforts are made to improve the expression efficiency, such as exploring different promoter to regulate the expression of heterologous proteins on the surface of spores [36, 37], selection of an appropriate anchor protein and peptide linker according to structural and functional properties of heterologous proteins [38].

In conclusion, our study indicates that OmpC can be successfully expressed and displayed on the surface of *B*. *subtilis* spores by using CotC as a fusion partner. Oral immunization in mice with recombinant spores can induce both antigen-specific systemic IgG and mucosal SIgA responses. Moreover, a significant level of protection against lethal challenge with *S*. Typhimurium is also detected in mice orally immunized with recombinant spores. Overall, these results suggest that *B*. *subtilis* spores have great potential as an attractive delivery system for heterologous antigens to the mucosal site of infection.
